# Beyond All Splits: Envisioning the Next Generation of Science on Mindfulness and Compassion in Schools for Students

**DOI:** 10.1007/s12671-022-02017-z

**Published:** 2022-11-11

**Authors:** Robert W. Roeser, Mark T. Greenberg, Tyralynn Frazier, Brian M. Galla, Andrei D. Semenov, Michael T. Warren

**Affiliations:** 1 Department of Human Development and Family Studies, The Pennsylvania State University, 119 Health and Human Development Building, University Park, PA 16802, USA; 2 Center for Contemplative Science and Compassion-Based Ethics, Emory University, 1599 Clifton Road NE, Atlanta, GA 30322, USA; 3 Department of Health and Human Development, School of Education, University of Pittsburgh, 3420 Forbes Avenue, 5th Floor, Room 534, PA 15260 Pittsburgh, USA; 4 Institute of Child Development, Carmen D. & James R. Campbell Hall, University of Minnesota, 51 E River Parkway, Minneapolis, MN 55455, USA; 5 Human Early Learning Partnership, The University of British Columbia, 2206 E Mall, Vancouver, BC V6T 1Z3, Canada

**Keywords:** Mindfulness, Compassion, Schools, Education, Human development, Students

## Abstract

**Objectives:**

This paper describes the emergence of the scientific study of mindfulness in schools; summarizes findings of experimental research on the impacts of school-based mindfulness programs (SBMPs) on student outcomes in prekindergarten, primary, and secondary school settings (ages 4–18 years); discusses scientific limitations and wider critiques of this work; and offers suggestions for future research.

**Methods:**

Public data are used to describe the emergence of science on SBMPs, the foci of this research, and the academic disciplines contributing to it. A narrative summary of scientific findings regarding the impacts of SBMPs on students, and critiques of this work, is also presented.

**Results:**

Research is increasing and is primarily psychological and prevention-oriented. Evidence shows SBMPs can enhance students’ self-regulation abilities, but SBMPs’ impacts on other student outcomes at different ages are equivocal. The current research has significant limitations, and these, alongside wider critiques of the work, suggest important directions for research.

**Conclusions:**

In the next generation of science, we suggest (a) *improving* the experimental research; (b) *expanding* developmental research; and (c) *re-envisioning* assumptions, theories, and methods in research to go “beyond all splits” towards a non-dualistic and relationally, culturally, contextually, ethically, and developmentally grounded science on mindfulness *and* compassion for students in schools.

During the past two decades, there has been a significant increase in the creation and implementation of school-based mindfulness programs (SBMPs) for students in preschool primary, and secondary school settings (ages 4–18 years of age). Data on the true reach of SBMPs remains elusive, but several educational organizations have previously indicated their reach extended to millions of youth and more than 50,000 educators worldwide (www.mindfulschools.org). The appeal of mindfulness programming for students has risen in conjunction with greater emphasis on social and emotional learning (SEL) in schools, as well as extensions of work in SEL to include the cultivation of students’ attentional, systems-thinking, and ethical skills alongside their social-emotional and academic ones (e.g., Goleman & Senge, 2014; UNESCO, 2019). There are now scores of school-based mindfulness programs in nations across the world that aim to teach students how to focus attention and be mindful in order to regulate stress; to reduce anxiety, sadness, and impulsivity; to improve health and well-being; to get along with others and act ethically; and to learn more and do better in school. And as the number of such school-based programs available to students has increased, so too have the number of scientific studies that assess their causal impacts on student outcomes.

In this paper, we reflect on this emerging field of applied science and on what we have learned and what is yet to be learned. The paper includes four parts: (1) a brief description of the emergence of the practice and science of mindfulness in schools for students; (2) a summary of what we have learned scientifically, and more specifically, experimentally, in the past 15 years regarding the causal impacts of SBMPs on child and adolescent student outcomes in educational settings; (3) a summary of scientific and humanistic critiques of this work; and based on these; (4) a set of suggestions regarding the next generation of science on mindfulness *and* compassion in schools for students that emphasize synthesis, wholeness, and “going beyond all splits” in research assumptions, theories, and methods in the future.

For instance, we propose a need to go beyond the split between research on mindfulness vs. compassion in schools and instead focus on an integrated approach to studying mindfulness *and* compassion in schools in future research given the conceptual overlap in these constructs (e.g., Roeser & Eccles, 2015), and the fact that both mindfulness and compassion practices are sometimes taught in the same school-based programs (e.g., Broderick, 2021). We also propose a need to go beyond dualistic (“split”) and mechanistic assumptions in research on mindfulness that theoretically and methodologically splits persons from contexts, minds from bodies, and thinking from feeling, in the search for causal understandings of what produces mindful and compassionate persons. Like others, we propose that mindfulness is not located in the head, nor in a manualized curriculum, but is something that emerges organically between individuals who are engaged in reciprocal social interaction and shared cultural activities and practices (e.g., Thompson, 2017). Relationships, interactions, and shared activity over time are hypothesized to be the heart of human development, including the development of mindfulness and compassion (see Bronfenbrenner & Morris, 2006). Thus, our proposal is that it would be beneficial to go beyond the conceptual splitting of persons from environments (and other people) in favor of focusing on relational constructs (e.g., persons in interaction in settings, embodied minds in activity, epigenetics), the concept of time (e.g., moments, stages, lifetimes), and related methods and measures in future research on mindfulness and compassion. We return to these suggestions for research at the end of this article.

## Emergence of Science on Mindfulness in Schools for Students

Beginning around the year 2000, there was a rapid increase in funded research grants and published research studies on mindfulness in the medical and clinical sciences (Bernstein et al., 2019). This period was characterized by the increasing use of mindfulness practices in healthcare and mental health settings to address physical and mental health problems, and thus the framing of mindfulness as a clinical-therapeutic approach to alleviating suffering became a key characteristic of the emerging science of mindfulness generally (Davidson & Dahl, 2018; Van Dam et al., 2018). Gradually, the new millennium saw a wider cultural mainstreaming of mindfulness in many countries (Kucinskas, 2018). Mindfulness was becoming more prevalent in both traditional and social media, and was beginning to be practiced in various sectors of society beyond medicine and mental health, including the human service professions, professional sports, business, and even government and the military (Boyce, 2011; Ryan, 2012).

Interest in developing school-based mindfulness programs for students and educators in schools also emerged in the early 2000s (e.g., Garrison Institute, 2005). A search of the phrases “Mindfulness in Education” and “Mindfulness in Schools” from 1990 to 2019 shows such the use of these phrases in all published English-language books has become more frequent, especially since 2000, in a trend that continues today (e.g., Supplemental Figure S1). In addition, organizations devoted solely to teaching mindfulness in school settings to students arose (e.g., MindUp: https://mindup.org; Mindful Schools: https://www.mindfulschools.org). In Great Britain in 2009, a gradual effort evolved in which SBMPs for students were implemented on a large national scale as a universal prevention and mental health promotion strategy (e.g., Mindfulness in Schools Project (MiSP): https://mindfulnessinschools.org/about/). Mindfulness apps have also become part of the mindfulness in schools landscape, with Headspace, for example, offering free access for K-12 educators (https://www.headspace.com/educators). In sum, the practice of mindfulness in schools has expanded considerably over the last 15–20 years.

Around 2005, researchers, educational scholars, and practitioners interested in mindfulness in education were beginning to meet and to build scientific networks and research partnerships. These networks began to create a new subfield of science and educational practice (Garrison Institute, 2005; Meiklejohn et al., 2012; MLERN, 2012; Roeser & Eccles, 2015; Roeser & Zelazo, 2012; Weare, 2013). Theoretical papers were written on the putative benefits of mindfulness in early childhood (Zelazo & Lyons, 2012), childhood and adolescence (Greenberg & Harris, 2012), and for educators (e.g., Jennings & Greenberg, 2009; Roeser et al., 2012). By around 2015, well-designed experimental studies were beginning to be published on the impacts of SBMPs on students during childhood (e.g., Schonert-Reichl et al., 2015) and adolescence (e.g., Kuyken et al., 2022; Raes et al., 2014); and on the impacts of mindfulness programs for school teachers (e.g., Jennings et al., 2017). By 2022, one of the largest cluster randomized trials of SBMPs in the world had been conducted in the UK with over 80 schools and 8000 adolescent students (MY Resilience In ADolescence or MYRIAD project: https://myriadproject.org; Kuyken et al., 2022).

**Fig. 1:**
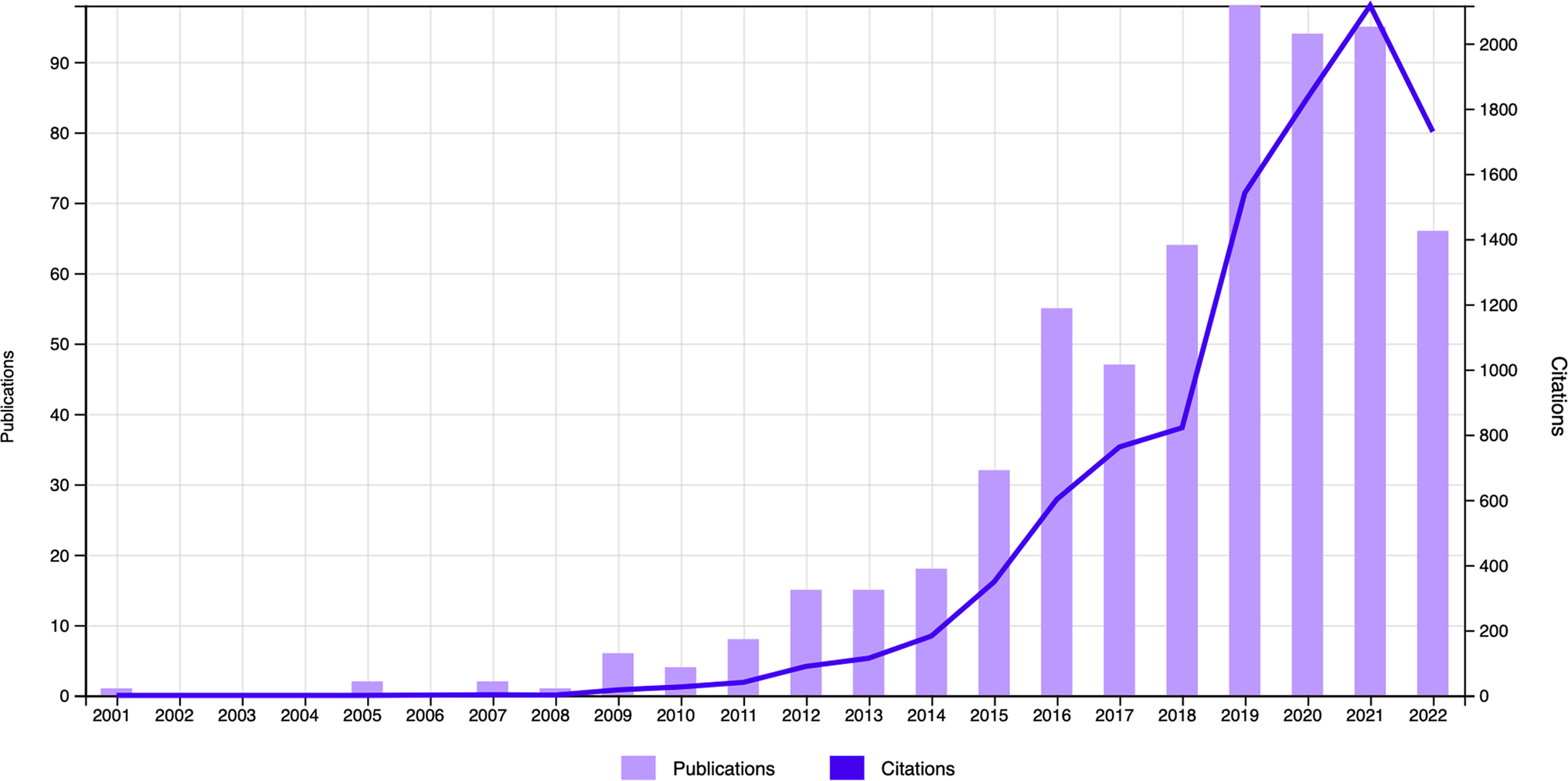
Research publications and citations on mindfulness, schools, and students from 2000 to 2022. Notes: *N* = 623 articles, *n* = 10,234 citations from Web of Science search. Search terms across “All Fields” included “mindfulness, schools, students.” We excluded from the search research articles on the “Topics” of “yoga,” “college,” and “medical” to focus on mindfulness and not yoga, and children and adolescents and not early adults. We also excluded articles from disciplines seemingly far from education or those perhaps related to educators (e.g., “Web of Science Categories”—“Heath Care Sciences and Services” and “Public, Environmental and Occupational Health”). This yielded *n* = 623 articles. An inspection of the constituent top 20 articles in the list confirmed their focus on “mindfulness, schools, students” during childhood and adolescence

An examination of published science articles and citations of articles on the topics of “mindfulness, schools, and students” in the Web of Science database revealed 623 published papers from the period 2000–2022. Data show that from 2000 to 2010, there was a rather slow increase in research volume, but after 2014, there was an uptick in the number of articles and citations that continues through today (see Fig. 1). Furthermore, data analytics from these 623 publications reveal that the top 5 disciplines accounting for 97% of the research are as follows: education and educational psychology (38.2%), clinical psychology and psychiatry (35.6%), other disciplines of psychology (12.5%), and developmental psychology (10.1%).

In sum, the science on mindfulness in schools has emerged only recently—during the past 10–15 years—and is still rather small in volume and scope. The science has also lagged behind the implementation and spread of SBMPs for students and educators in schools across the world. Currently, it appears that clinical, psychiatric, psychological, and educational perspectives and research methods are well represented in the scientific knowledge base on mindfulness in schools for students. On the other hand, similar to the wider science on adult mindfulness (Van Dam et al., 2018), the science of mindfulness in schools for students does not yet appear to be very developmental in nature. In the next section, we examine the current state of evidence for SBMPs to evaluate what we know and do not yet know currently about the impacts of such programs on students during the preschool, primary, and secondary school years (ages 4–18 years).

## Experimental Evidence Regarding the Impacts of School-Based Mindfulness Programs on Student Outcomes

In 2020, a research brief was prepared for the Robert Wood Johnson Foundation that summarized the current state of science on the impacts of SBMPs on students - “Mindfulness in schools: Evidence on the impacts of school-based mindfulness programs on student outcomes in P–12 educational settings” (Roeser et al., 2022). The brief was designed to be a summary of the science for educational practitioners, leaders, and policy makers. It evaluated and summarized the experimental research evidence regarding whether SBMPs, delivered in prekindergarten (pre-k or preschool) through secondary school settings, impacted the following student outcomes: (a) mindfulness and self-regulation skills; (b) mental health (absence of internalizing and externalizing distress, presence of well-being), (c) physical health; (d) relationships with other people and nature; and (e) school behavior and learning.

## Selection and Description of Research Studies

Given that experimental studies are an important part of the educational and prevention-in-schools research landscapes (Raudenbush & Schwartz, 2020), the research brief focused solely on experimental studies that used randomized control or quasi-experimental study designs to ascertain causal program impacts on students. Based on a series of research reviews conducted by scholars in the field (Baelen et al., 2019; Carsley et al., 2018; Felver et al., 2016; Klingbeil et al., 2017; Maynard et al., 2015; McKeering & Hwang, 2019; Semple et al., 2017; Weare, 2019; Zenner et al., 2014; Zoogman et al., 2015), as well as the authors’ own review of the research, Roeser et al. (2022) identified 54 studies for consideration. These studies were conducted on 36 different SBMPs (there were multiple evaluations of some programs) and included over 13,000 students.

Studies were selected for inclusion based on the following criteria: (1) the program reported an explicit mindfulness component and was taught to students during the school day; (2) the study was published in a peer-reviewed scientific journal in English; (3) the study used an experimental design that included a randomized or matched-comparison group of students against which to assess program impacts; (4) the studies included pre/post measures; and (5) the treatment and control groups consists of 30 total participants or more to ensure reliable estimation of program impacts between groups. A third of the research studies included students from primarily low-income backgrounds, a third included a majority of students from immigrant or underrepresented racial/ethnic groups, and 89% of studies evaluated a universal SBMP rather than a targeted program (e.g., programs offered only to a subset of students with identified risk factors). The lack of inclusion of studies in languages other than English or studies of school-based programs for students focused primarily on themes of kindness, empathy, self-compassion, or compassion given the nascent nature of this work (e.g., Jazaieri, 2018; Lavelle et al., 2017) is important to note here and is discussed below in future directions for research below.

## Nature of the School-Based Mindfulness Programs

Roeser et al. (2022) briefly characterized the nature of the 36 different SBMPs that were evaluated in the selected research studies (see Brief Appendix). Each program was coded in terms of its origins and structure (e.g., adapted Mindfulness-Based Stress Reduction (MBSR), novel, brief), the program facilitator (e.g., external instructor, classroom teacher), whether the program included home practice, and the total estimated time/dose of the program. Descriptively, approximately 30% of the SBMPs were adaptations of multicomponent mindfulness programs for adults (e.g., Mindfulness-Based Stress Reduction; Mindfulness-Based Cognitive Therapy); about 50% were novel (newly developed and incorporating content from neuroscience, social and emotional learning, and positive psychology); and the remaining 20% were characterized by the use of brief practices only without a formal program. Over 50% of the programs were delivered by external facilitators, about one-third were delivered by teachers, and the rest were delivered by both (with some studies not reporting this information). Homework was encouraged in about one-third of programs but few required it (less than 10%), although most studies did not report this information. In terms of program time/dose, approximately 25% were 6 h or less; about 50% were 6–15 h in total; and about 25% were more than 15 h of total school time. The programs were highly variable in the number of sessions per week, the duration of the program in terms of weeks, and the amount of time spent during each session and between sessions.

Based on the coding of studies, Roeser et al. (2022) found that the most commonly assessed student outcomes of these programs were self-regulation abilities (and to a lesser extent, students’ mindfulness skills), followed by internalizing distress (e.g., symptoms of anxiety, depression), externalizing distress (e.g., symptoms of anger, aggression), and psychological well-being (e.g., positive emotion, optimism). Students’ physical health, relationships with other people and the natural world, and school outcomes (e.g., grades, conduct) were less frequently studied. Self-regulation abilities were the most assessed outcome examined in preschool studies; distress was assessed more than well-being generally and only in primary and secondary schools; and there were no studies that looked at school grades or behavior in high school students.

## Experimental Evidence Regarding Program Impacts on Students

In terms of program impacts, the brief reported tentative positive evidence for the impact of SBMPs on improvements in students’ self-regulation abilities, reductions in symptoms of anxiety and depression, and improvements in their physical health and relationships with others. Little consistent evidence was found that SBMPs reduced students’ anger or aggression, or improved feelings of well-being. It was determined that further study is needed to adequately assess program impacts on students’ school behavior and performance. Overall, the brief authors concluded that “What we found was promising evidence that SBMPs can positively impact students’ mindfulness and self-regulation skills, reduce students’ internalizing distress, and improve students’ physical health and healthy relationships” (Roeser et al., 2022, p. 13).

Since the preparation and publication of the research Brief, new data have been published from a large and rigorous randomized control trial of a SBMP for early adolescent students. The MYRIAD (My Resilience in Adolescence) Trial included more than 8,000 adolescents (age range = 11 to 14 years) from 85 schools in the United Kingdom. Youth were randomly assigned (at the school level) to receive either a 10-lesson SBMP (called “.b”) or to continue with their schools’ existing social-emotional learning curricula. In a paper detailing the treatment main effects on primary and secondary outcomes, assessed at immediate post-test and one-year follow-up, Kuyken and colleagues (2022) reported no benefits of the SBMP on students’ externalizing distress (e.g., conduct problems) or psychological well-being. These findings were generally consistent with the conclusions of the research Brief. However, the trial also found no benefits for early adolescents’ self-regulation (executive function) or mindfulness skills, internalizing distress (depressive symptoms, anxiety), physical health (e.g., drug and alcohol use), or healthy relationships (e.g., prosocial behavior), These conclusions were inconsistent with the conclusions of the Brief. There are, of course, important caveats that prevent direct comparisons between the conclusions of the Brief and the MYRIAD Trial (e.g., the Brief did not disaggregate results of SBMPs for early adolescent students only). Nevertheless, the MYRIAD trial does call into question the utility of universal SBMPs to support mental health in early adolescents - something we discuss below. In the next section, we describe some of the methodological limitations in the current research base that were noted in the Brief.

## Limitations of Experimental Research Reviewed

Roeser et al. (2022) noted 4 main limitations in the reviewed research. First, they noted methodological limitations in the designs of existing experimental studies. The 54 studies generally did not report power analyses to determine sample sizes needed for detecting significant differences in outcomes due to treatment. Studies that fully randomized students to condition; employed active control conditions instead of business-as-usual controls; or used multilevel designs to account for the nesting of students in classrooms and schools, were not common. The collection of follow-up data on students beyond the post-program assessment period was rare. Thus, issues of power, randomization, active comparison conditions, multilevel nesting in analyses, and collection of longterm follow-up data were all noted as methodological limitations in existing studies.

The second limitation noted was a relative lack of developmental conceptualization and measurement of mindfulness in students across different ages, with different methodologies (e.g., Goodman et al., 2017). Mapping and measuring the hypothesized top-down (e.g., executive attention, executive function) and bottom-up (e.g., curiosity, security) elements of mindfulness in reliable and valid ways across development was under-developed in the work reviewed.

The third limitation noted had to do with the conceptualization and description of the programs and the contexts in which they were implemented. Though many of the reviewed studies did collect implementation data, this data was not always clearly and coherently organized and presented in relation to program impact data via logic models. The need for greater clarity of program descriptions in future research in terms of (a) the *core program components* and *components of the wider implementation support system* for the program in the school; (b) the *participants* (those who deliver, those who receive the programs); and (c) the *multilevel contexts* of the school, neighborhood, district, and larger community context in which such programs were embedded was noted (see Baelen et al., 2022; Durlak & DuPre, 2008). Thus, we need to know more about how key program, school, instructor and student characteristics mediate program impacts on student outcomes.

The fourth limitation noted was that few of the studies reviewed offered or tested developmental hypotheses regarding the moderation of program impacts by characteristics of students, instructors, or the classroom and school settings. Research on social and emotional learning programs for students suggests factors at each of these levels are important moderators of program impacts (Bierman et al., 2010; Cramer et al., 2021; McCormick et al., 2015). The lack of attention to how different individual characteristics and different setting characteristics condition program impacts was noted. It is important to note that the MYRIAD project mentioned above was designed to address many of these limitations (see Kuyken et al, 2022).

Due to these four limitations, Roeser et al. (2022) concluded that “[w]e still know relatively little about which kinds of programs and practices, for which kinds of outcomes, for which students, at which ages, work best” (p. 13). In sum, existing experimental evidence suggests some promising positive impacts of SBMPs on student-level outcomes, but also null findings that require scrutiny. In addition, an understanding of context and development is relatively under-developed at this time in the experimental research. And so, despite some evidence of promise and little evidence of harm (see also Filipe et al., 2021), the brief revealed many unanswered questions, scientific limitations, and a limited ability to translate the science in ways relevant to the concerns of educational policy makers, leaders, and practitioners at this time.

## Wider Critiques of Mindfulness in Education Research

Beyond these scientific limitations, broader critiques of the practice and science on SBMPs for students have been raised by scholars from philosophy of education, contemplative studies, and philosophy of science perspectives. We see these broader humanistic critiques, alongside scientific ones, as important to consider when conceptualizing the next generation of science on mindfulness and compassion in schools.

## Academic Discourses in Research on “Mindfulness in Education”

The first critique of the research on mindfulness in schools for students comes from a philosophy of education perspective, and is based on a discourse analysis that reveals a “split discourse” in the academic research community with regard to mindfulness in education. Ergas and Hadar (2019) analyzed the academic discourse in publications about mindfulness in education from 2002 to 2017 in terms of three main themes: (a) the *framing* of the origins and nature of mindfulness programs and practices in education, (b) the intended educational *aims of such practices,* and (c) associated *pedagogies/methods of teaching these practices* (see Table 1). Their analysis revealed what they viewed as a predominant academic discourse in the research that they called a “mindfulness *in* education” approach. This discourse was contrasted with a second, less recognized academic discourse they termed “mindfulness *as* education” (Ergas & Hadar, 2019—see Table 1).

According to Ergas and Hadar (2019), mindfulness *in* education discourse frames mindfulness in educational settings in terms that are secular, psychological, individualistic, and therapeutic in nature. In the major discourse, mindfulness programs and practices are described in terms that are “effects-oriented,” involving the implementation of short-term “interventions with concrete measurable effects” such as “well-being, physical and mental health...social emotional learning and the enhancement of cognitive functions (p. 757).” The term “intervention” is used often, a term that has its origins in the medical sciences and lends itself to economic (cost-effectiveness) analyses (Ergas, 2019). In terms of pedagogical methods, there is little discussion in this discourse regarding the integration of mindfulness practices into the wider curriculum and culture of the school. Mindfulness is a tool for cultivating individual well-being within the existing school system (Ergas, 2019). Ultimately, Ergas (2019) is critical of the educational value of this approach, seeing it as serving, rather than transforming, the existing educational system by being therapeutic, individualistic, instrumental, and economic in educational frame, aim, and method. Instead, he argues for a “mindfulness *as* education” discourse that is more culturally and ecologically grounded and focused on personal *and* social transformation—specifically, the transformation of educational settings themselves (see Table 1; Ergas, 2019).

The predominant discourse identified by Ergas and Hadar (2019) has been similarly criticized by scholars from contemplative studies. Some have raised concerns about how the individualistic framing can promote a view among novices that contemplative practices are essentially private, engaged in alone, and about self-help (Condon & Makransky, 2020). This can be contrasted with an approach that reclaims the relational starting point of such practices, and positions them as inherently relational in nature; learned and engaged in with others in a supportive community, and aimed at cultivating belonging and caring for others, self, and world (Condon & Makransky, 2020). Similarly, the positioning of mindfulness as a kind of individualistic, economic commodity for personal gain, one that can be accrued through practice within a wider competitive and consumptive culture (e.g., “McMindfulness”), has been criticized (Purser & Loy, 2013). Others have discussed how individualistic framing of mindfulness may lead to an over-emphasis in programs on the individual causes of human suffering within the mind, and thus promote the “privileging of highly individualized descriptions of suffering and health, thereby eschewing social and systemic causes of suffering” (Lavelle, 2016, p. 233). Instead, scholars like Forbes (2016) point to a need to promote the social aims of mindfulness practice for students by embedding such practice “within critical, integral programs that uncover and resist dominant ideologies and institutions in which we swim and to consciously help us heal and create new relationships that work towards optimal personal development and universal social justice” (p. 355).

In sum, critiques exist suggesting that the predominant academic discourse today in research on mindfulness in schools frames mindfulness and its aims in education in ways that are too individualistic, universalistic, economic, and instrumental. The framing of mindfulness, such critiques suggest, is not sufficiently focused on mindfulness as a way of education and life, ethical conduct, and social transformation (e.g., social responsibility and social engagement). The concern is that the “combined effects of secularization, scientific measurement and commodification deracinate the practice from its ethical grounds and erode its transformative potential” (Ergas & Hadar, 2019, p. 2). Broadening the framing and evaluation of mindfulness in schools in future research and practice to be more relationally, culturally, contextually, ethically, and developmentally grounded is implicated by such critiques. This broader framing is what Ergas and Hadar (2019) refer to as a “mindfulness *as* education” discourse (see Table 1).

## Meta-Theories in Research on Mindfulness for Students in Schools

Another critique from philosophy of science perspectives focuses on the limitations of certain dualistic and mechanistic meta-theoretical assumptions and views underlying current research on mindfulness in Contemplative Science generally, and in education specifically (e.g., Roeser, 2016; Thompson, 2017, 2020). Meta-theories have been described as a set of (often implicit) interdependent principals that serve to describe and prescribe “what is meaningful and meaningless, acceptable and unacceptable, central and peripheral, as theory – the means of conceptual exploration – and as method – the means of observational exploration – in a scientific discipline” (Overton, 2007, p. 154). Meta-theories transcend “theories and methods in the sense that they define the context in which theoretical and methodological concepts are constructed,” (pg. 154), providing an implicit evaluative frame in scientific research that functions to define what is central, meaningful, and acceptable to examine conceptually and methodologically.

Overton (2007) describes 2 major classes of meta-theories in the science of human development that are relevant here: the split and the relational. Split meta-theories “are based on Cartesian thought and tend towards reductionism and linear and additive organization. Such meta-theories divide the world into dichotomous either/or propositions (e.g., the real is mind, or body, or some additive combination of the two)” (p. 156). The root image of the human being in split meta-theories is often a clockwork or other machine metaphors, and thus recourse to the language of mechanisms (e.g., Merchant, 1980). On the other hand, relational meta-theories are “post-Cartesian” and aim towards holism, non-linear and non-additive organization, and synthesis. Relational meta-theories aim to “transform classically fundamental dichotomies into indissociable complementarities in the study of human beings through the relational principles of holism, identity of opposites, opposites of identities, and syntheses of wholes” (Overton, 2007, p. 156). Here, separating human functioning into the kinds of antimony described above (e.g., the body vs. the mind, genes vs. culture) is eschewed in favor of concepts aimed at synthesis. Thus, research attention is given to integrative topics such as individuals-embedded-in-multiple social contexts, to embodied minds, epigenetics, and so on as ways of re-envisioning seemingly fundamental opposites. The root image in relational meta-theories is often the cell or other organic/organismic metaphors that draw on meanings associated with living systems (e.g., Sameroff, 2010).

One philosophy of science critique of research on mindfulness in contemplative science and neuroscience today offered by Thompson (2017, 2020) concerns what can be considered a split individualistic and materialistic metatheory. The critique is that scientists too often frame the study of mindfulness in neurocentric terms, as something in the head, something brain-bound. This kind of framing, which assumes that the individual is separate (split off) from the world, can reinforce “selfish individualism” and the “McMindfulness” perspective discussed above by presenting mindfulness as a self-help commodity that can be acquired through privatized practice (Thompson, 2020). Instead, Thompson (2020) articulates a relational frame or meta-theory, the 4E enacted cognition perspective, in which the study of mindfulness involves a “whole embodied being embedded in the world” (Thompson, 2020, p. 12). From such a relational point of view, mindfulness is assumed to be the quality of a whole person acting in goal-directed ways in social contexts with others. Mindfulness is not assumed to be a just feature of the mind or the brain, or something “in the head.” It involves a coupling of the brain-body and world.

There are two implications of this view that Thompson draws out. First, a relational set of assumptions assumes a whole person, with agency, intentions, and motivations, when conceptualizing mindfulness. In contrast, a split view frames a person as a mind vs. body. In the first side of the assumed dichotomy, mindfulness is reduced to cognitive and emotional skills; in the second, it is reduced to neural patterns of brain activation. Thompson (2017, 2020) is critical of a further materialistic reduction in this assumptive frame, the mind as epiphenomenal to the brain, and therefore resultant notions like the mindful brain (Siegel, 2007). In these instances, the relational notion of mindfulness as a quality of a whole person, with agency, intentions, and motivations, interacting in social and physical worlds with others to address developmentally-relevant needs and life priorities, is peripheral.

**Fig. 2:**
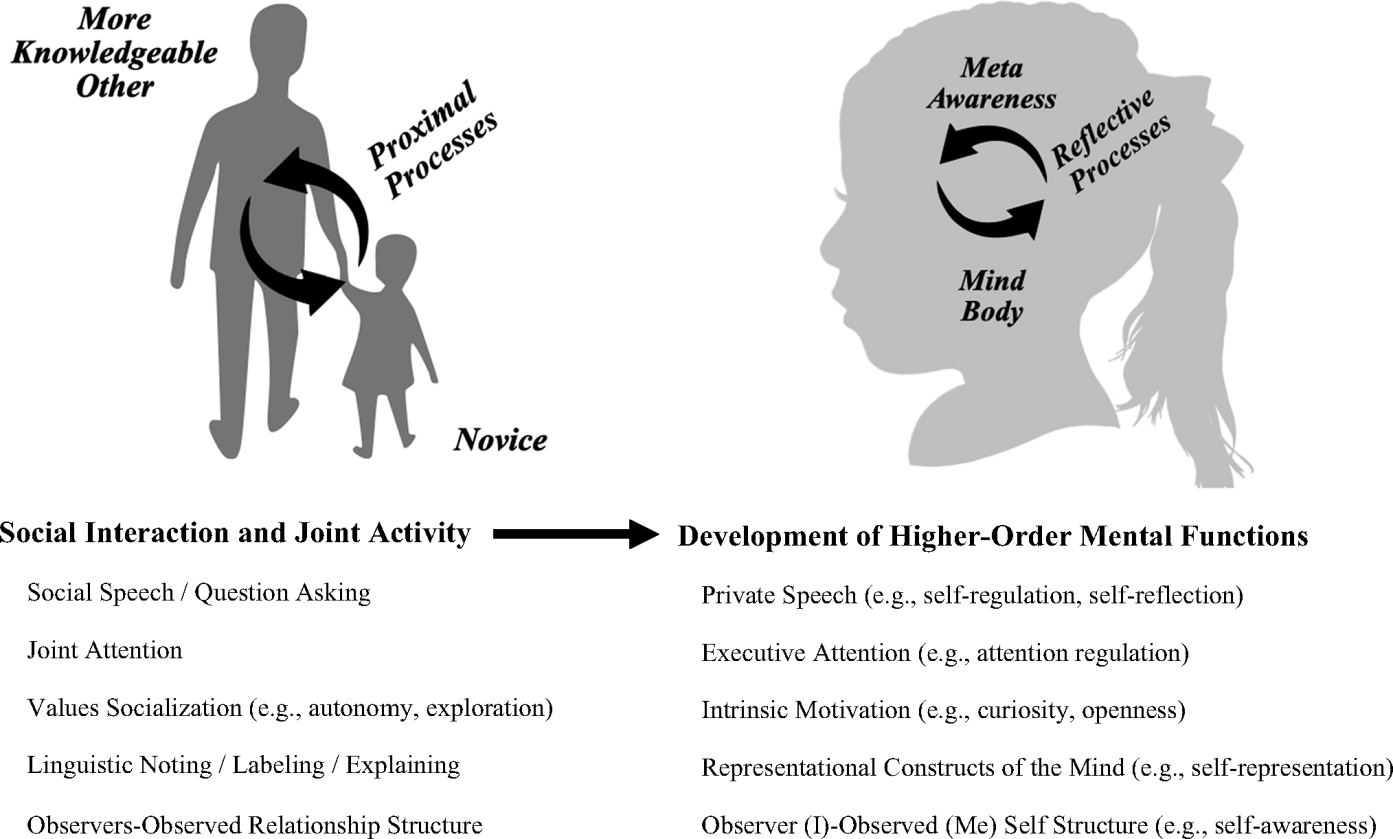
Sociocultural, developmental-relational perspective on higher-order mental functions like mindfulness

The second, related implication that Thompson (2017, 2020) draws out in his critique is that mindfulness is fundamentally a social practice: learned from others, and practiced in relationship to others and lived sociocultural worlds. In terms of origins, a relational meta-theory assumes that social relationships, social interaction, and shared activities between children (e.g., novices) and adults (e.g., experts) are key processes in how one learns to become a mindful or compassionate person (see Fig. 2). One learns these qualities in a cultural community of practice with so-called more knowledgeable others. This represents a sociocultural, developmental-relational view of learning and human development. Development here is about interaction and activity with others, and involves the intergenerational scaffolding of higher-order mental constructs like mindfulness through relationships and attunement, modeling and verbal instruction, and shared attention—what might also be cultural socialization or the routines of teaching and learning (see Fig. 2; Rogoff et al., 2018; Sameroff, 2010; Tharp & Gallimore, 1991). From such a view, as the well-known phrase by Vygotsky (1978) stated it:

Every function in the cultural development of the child appears on the stage twice, in two planes, first, the social, then the psychological, first between people as an ‘inter’ mental category, then within the child as ‘intra’ mental category. This pertains equally to voluntary attention, to logical memory, to the formation of concepts, and to the development of will. All higher functions originate as actual relationships between people (p. 57).

From a sociocultural, developmental-relational perspective, the development of mindfulness and compassion (and other higher-order psychological processes);

...takes place through processes of progressively more complex reciprocal interaction between an active, evolving bio-psychological human organism and the persons, objects, and symbols in its immediate external environment. To be effective, the interaction must occur on a fairly regular basis over extended periods of *time*. Such enduring forms of interaction in the immediate environment are referred to as *proximal processes*. (Bronfenbrenner & Morris, 1998; p. 996).

Furthermore, social interactions and proximal processes that provide a relative fit (rather than mismatch) with the needs and capacities of the developing person are thought to instigate, rather than forestall, motivation to learn and the development of higher-order mental processes (e.g., Eccles & Roeser, 2016). From a relational perspective, it is theorized that:

an intricate relation between inner (cognitive and emotional) development and a stimulating and encouraging environment exists from the beginning of life, so that no stage or developmental crisis could be formulated without a characterization of the mutual fit of the individual’s capacity to relate to an ever expanding life-space of people and institutions, on the one hand, and on the other, the readiness of people and institutions to make him [sic] part of an ongoing cultural concern” (Erikson, 1970; p. 754).

An illustration of how one might conceptualize the origins of mindfulness from this assumptive and theoretical perspective is presented in Fig. 2. It depicts the elements of mindfulness as hypothetically developing through attuned interactions between a caring and mindful adult (e.g., a more knowledgeable other), a secure and curious child (e.g., the novice), and the cultural practice of joint attention in everyday life (e.g., Shaver et al., 2007). Within a context of care, it is hypothesized that the practice of joint attention over time in everyday life is a key proximal process shaping the child’s curiosity, attention, noting and linguistic labeling, verbal understanding, reflection, and, eventually, meta-awareness. The hypothesis is that through social interaction with an adult, the child develops meta-cognition, the capacity to “examine one’s own thinking from the perspective of the other and thereby re-describe one’s own cognitive representations of the world” (Thompson, 2007; p. 405). This social process is theoretically related to reflection and the reiterative reprocessing of representations of self, others, and world (Zelazo, 2015), as well as the related capacity to observe one’s mind from the stance of a witness (Engler, 1984).

The notion that mindfulness is learned socially through interaction with others has implications for conceptualizing an individual’s private meditation experience as well. From a relational point of view: “[m]editative introspection is not inner perception of an independent and preexistent, private mental realm; rather, it is meta-cognition (internalized social cognition) of socially constituted experience” (Thompson, 2017, p. 60). From a relational perspective, mindfulness practice, whether done in a group or alone, is a social practice. Both the observer-observed structure of consciousness, and the mental contents observed during practice, are socially-constituted.

Dualistic and mechanistic assumptions, rather than relational and organismic ones, characterize to some extent the existing experimental research on mindfulness in schools. As described above, the first generation of experimental research focused largely, and understandably, on the general question “did the mindfulness program (in the form of a manualized curriculum) work?” In the main, the research to date has not focused on integrating assessments of what happened inside the “black box” of the program and its implementation with the study of program impacts (see Baelen et al., 2022). Thus, we do not yet have a complex and coherent relational, contextual, and developmental understanding of how and why SBMPs for students work or not. Rather, we have a kind of mechanical view of how a manualized curriculum on mindfulness impacts students or not, without much elaboration on how teachers and students, with their qualities, agency and intentions, interact and engage with the curriculum together to produce outcomes (or not). In this case, the critique is that mindfulness training is being conceptualized in a relatively flat way-almost as synonymous with a manual or an educational curriculum. Rather, we see it as fruitful to see a mindfulness curriculum as something a student learns largely through the efforts of a competent teacher who is able to bring that curriculum and set of practices into the child’s life in a way that is relevant, vitalizing and addresses the child’s developmental needs (e.g., Dewey, 1902). Here, we conceptualize mindfulness not only as a social practice, but an intergenerational, pedagogical, and developmentally-attuned social practice.

In sum, split meta-theories can lead to debatable conclusions like mindfulness is “in the head” or mindfulness is a treatment that is “in a manual or curriculum.” As we have been suggesting, a relational meta-theory goes beyond dualistic efforts that serve to theoretically separate, and often flatten, features of individuals or social environments. A relational meta-theory is not an effort to shift from individual to contextual approaches (and back again) in efforts to understand the development of mindfulness (see Sameroff, 2010). Rather, it is an effort to go beyond all splits in assumptions to advance scientific understanding (Overton, 2007). Whether or not these kinds of critiques and alternative assumptions and hypotheses they lead to prove accurate, they illustrate how a shift from split to relational meta-theoretical assumptions shifts the theoretical and methodological focus towards thinking about whole persons—their agency, motivations, and stage-specific developmental needs (as well as how well their social environments address such needs). Such a perspective also serves to orient theory and method towards contexts, relationships, social interaction, shared activity, and cultural practices in the development of mindful and compassionate children and adolescents. These issues are precisely those that need attention in the research currently, and that are made peripheral by split views in which mindfulness is understood as something in the head or in a manual/curriculum. In the final section, we provide a summary and propose suggestions for future research based on our review.

## Summary and Suggestions for the Next Generation of Science on Mindfulness and Compassion in Schools

Our review reveals significant issues and questions at the interface of research and practice on mindfulness and compassion in schools that require attention. The spread of mindfulness and compassion programs in education is growing, as is the scientific study on school-based programs. We characterize the research as primarily psychological and educational, and less so relational and developmental, in nature at this time. The number of experimental studies is small. A review of a subset of these studies revealed that despite evidence of promise, especially for attention and self-regulation abilities, and little evidence of harm, definitive conclusions about the impacts of SBMPs on students in preschool, primary, and secondary school settings are limited by the small number of studies and their design features. As a consequence, there are many things, some fairly basic, that we still do not yet know (e.g., how can mindfulness best be understood and measured at different ages in childhood and adolescence?). At the same time, philosophy of education critiques focus on the widespread individualistic, psychological, and instrumental framing of mindfulness in education, and suggest a more relational, ecological, ethical approach to mindfulness in schools would be valuable (see Table 1). Philosophy of science critiques take aim at the scientific assumptions used to frame research on mindfulness by noting these are often “split” (e.g., dualistic), mechanistic, and non-developmental. Such assumptions tend to obscure attention to the whole person and their agentic role in their own development, as well as the nature of contexts, social relationships, and shared activity and practice in the production of mindful and compassionate persons (see Table 2). In the aggregate, the current state of the field, as we understand it, leads to three main suggestions for future research on mindfulness and compassion in schools.

## Re-Envisioning Approaches and Assumptions

First, this review raises fundamental questions about the assumptions and frames underlying both practice and scientific approaches to the study of mindfulness and compassion in schools. The value of reflection and research on discourse practices and meta-theories in a scientific field is that it tends to make salient the evaluative frames, words, and images that shape the way we think about, implement, and scientifically evaluate mindfulness and compassion programs in schools. Thus, reflection on assumptions and frames is a key part of the evolution of a scientific field. The critiques summarized here, which propose that the current framing of mindfulness in education is too individualistic, instrumental, mechanical, and dualistic, and not focused enough on the relational ecology of children in schools, or ethics and social change, seem important to consider in the next generation of research (see Tables 1–2).

Much of our review was focused on experimental studies of SBMPs for students implemented in individual classrooms over a relatively short time horizon. The data show the impacts of such programs were promising, through equivocal; and new data from a rigorously designed study has raised questions about the effectiveness of a universal, school-based approach, at least with early adolescents in the UK, works (Kuyken et al., 2022). One wonders, based on such findings, if a self-selective rather than universal approach, where such courses are offered to adolescents, for instance, as electives, might be an alternative way to deliver such programs to those who are interested and see them as relevant (see for instance: https://www.peaceinschools.org). In addition, the MYRIAD project raised important questions as to whether or not teachers *in general* are able to competently teach SBMPs (Crane et al., 2020).

Thus, as a field, we may need to re-envision our pedagogical and systems-change approach to bringing mindfulness and compassion into schools, and consider the ecology of the school as a whole as a primary unit of analysis in this work, not the single teacher in an isolated classroom. We know little about the implementation of mindfulness as a school-wide practice, where it is integrated into the culture and life of a whole school and involves both teachers and students (e.g., Sheinman et al., 2018). More qualitative, interpretative, and action research on implementation in whole schools are needed. In addition, there is strong evidence that mindfulness works for improving teachers’ occupational health and well-being, and accumulating research suggests that teacher programs also have impacts on how teachers interact with students in the classroom (see Klingbeil et al., 2017). More research is needed on how training educators can have indirect downstream consequences on student outcomes. Finally, it may turn out that universal or school-wide programs are only viable strategies in certain contexts, and not at scale, and that alternative contexts like retreats or camps are useful to study in the future (e.g., Galla, 2017). In sum, our review suggests it is a good time to reflect and re-envision assumptions about how mindfulness and compassion might enter education *pedagogically* and *organizationally* in the most fruitful way (e.g., Ergas, 2019), and be studied in the most fruitful way.

Our review also suggests that scientists need to adopt a set of scientific assumptions that match the kind of interpersonal and organizational complexity involved in teaching and learning such practices in school settings for children and youth. As over 100 years of debate and discussion revealed among developmental scientists, split assumptive meta-theories in the study of human development show scientific shortcomings with regard to understanding development by splitting apart processes that are actually interdependent and holistic in nature (e.g., Lerner & Overton, 2008; Zelazo, 2013). The either/or, split assumptive frame reduces the multiple, multilevel, dynamic processes that causally relate to the development of mindful persons into incomplete understandings at best. Such assumptions do not focus specifically on the proximal processes that interface between such presumed splits (e.g., persons vs. environments), processes that range across multiple levels from cells to society or neurons to neighborhoods in the development of qualities like mindfulness (e.g., Roeser et al., 2018; Zelazo, 2013).

A relational meta-theory that goes beyond all splits has led to a “new synthesis” in developmental science whereby a consensus and coherence of assumptions theories and constructs, and methods has ensued (Zelazo, 2013). Such a meta-theory leads to theories of mindfulness that conceptualize it first and foremost as an intergenerational social practice—as something being worked out with other people, in specific settings, through language and gesture, intergenerational relationships and joint activity around cultural practices (e.g., meditation, service), over a sustained period of time in the lifespan. Here, notions like the “fit” between the developmental and cultural needs of children and adolescents, and these kinds of programs and practices, become focal, as do related issues of motivation and engagement on the part of students to engage with such programs and practices (e.g., Walton & Yeager, 2020; Yeager et al., 2018).

We hope we have spurred reflection on meta-theories in this paper which will help to lead towards a new synthesis in Contemplative Science and the study of mindfulness and compassion in schools in the future around a meta-theory that is relationally, culturally, contextually, ethically, and developmentally grounded (see Thompson, 2020). Such a synthesis could heal the split that currently exists between the clinical and neuroscientific work with adults in Contemplative Science, and the research focused on children, adolescents, and parents and teachers in families and schools that Roeser and Zelazo (2012) called Developmental Contemplative Science. It might lead to closer integration in the science of mindfulness and compassion. Finally, re-envisioning meta-theoretical assumptions to be more relational might also focus both experimental and developmental research in schools more fully on whole persons and their identities, social relationships and interaction, cultural practices, and holistic school settings in shaping mindful and compassionate persons in different countries and school systems (e.g., Colaianne et al., 2019). These are the very issues that demand greater attention in the science currently, and could lead to new insights as to how to create mindful and compassionate communities of learning in schools in the future. In addition to re-envisioning assumptions and frames, we see a need for improvements in experimental research and the need for more developmental research in this area in the future. Given the complementarity of experimental and developmental approaches (see Table 3), we also hope to see greater cross-fertilization between these forms of research going forward (e.g., Bronfenbrenner & Morris, 2006).

## Improving Experimental Research

Based on our review, we also propose that *improving experimental research* on school-based mindfulness and compassion programs in the next generation of science is important. In part, the improvements that we recommend for experimental studies of programs in schools parallel suggestions made for the science of mindfulness in general (Davidson & Kaszniak, 2015; Dimidjian & Segal, 2015), and build upon work in the broader field of social and emotional learning (SEL) in schools around the study of program implementation (Durlak et al., 2011; Greenberg et al., 2017). In the first case, future studies of SBMPs might employ more power analyses, greater use of randomization to conditions, larger sample sizes, use of active controls, consideration of non-specific factors in intervention results (e.g., instructor motivation and enthusiasm), and collection of longer-term follow-up data. In addition, consistent with work in SEL, experimental students of program impacts need to integrate implementation science into the study of impacts to understand how and why programs work or do not work (Bryk, 2016; Durlak & DuPre, 2008; Greenberg et al., 2017), and to focus on the fit of programs within the wider ecology and cultures of schools (e.g., Roeser et al., 2022). These issues are explored in greater depth by Baelen et al. (2022).

Furthermore, more research on moderated program impacts is needed in terms of both student characteristics (e.g., student age, gender, ethnicity, race, disability status) and school characteristics (e.g., leadership, culture, support for program implementation). Addressing questions of moderation would improve future research by providing answers to questions of practical relevance for educators regarding program impacts for whom, for what outcomes, when (in development), and where (in terms of contextual settings). Relatedly, attention to issues of diversity, equity, and inclusion in program design and evaluation is critically important (e.g., Jagers et al., 2019).

More conceptual and measurement work is needed on assessing mindfulness, compassion, and their associations with other developmental outcomes (e.g., social competence, well-being, school learning) across childhood, adolescence, and adulthood. Creating new, multimethod, valid, and reliable measures of these key proximal outcomes, in ways that are sensitive to change due to development and intervention, is a critical priority (Roeser et al., 2018). Such measurement work would improve our understanding of the mediating roles that mindfulness and compassion play in program impacts, and would advance our understanding of how to measure these constructs as embodied by teachers or those who implement programs in schools (e.g., Rickert et al., 2019).

## Expanding Developmental Research

Our final suggestion based on our review is *expanding developmental research* on mindfulness and compassion during childhood and adolescence across diverse cultural, linguistic, national, and geographic settings in the next generation. Developmental science has three main aims with regard to individual development: (a) a description of continuity and change over time in personal characteristics (mind, body, behavior), within and across persons (and corresponding contextual conditions); (b) an explanation of how biological, social and cultural factors interact in the production of intra- and inter-individual continuity and change in these characteristics over time; and (c) the use of scientific descriptions and explanations for the optimization of human development. Optimization occurs through various intervention and prevention efforts aimed at modifying or preventing risk, disease and distress-producing conditions and processes; as well as through promotion efforts aimed at cultivating resilience, health, and flourishing-producing conditions and processes (e.g., Baltes et al., 1977).

Expanding basic developmental science to create a coherent description and explanation of the development of mindfulness and compassion in schools and other social contexts during childhood and adolescence would advance this area of science (see Roeser & Eccles, 2015; Spinrad & Eisenberg, 2017). A few examples of basic unanswered questions of this kind include the following: what are the elements that constitute mindfulness and compassion across development, and how and why do these elements differentiate and integrate across time? How might we best conceptualize and measure mindfulness and compassion across developmental time—as skills, dispositions, or identities? Are developmental changes in mindfulness best conceptualized as gradual and quantitative in nature (e.g., more and more re-iterative reprocessing and reflection—Zelazo & Lyons, 2012); more abrupt and qualitative in nature (e.g., a sudden shift to a “witness” perspective—see Engler, 1984), or both? Does mindfulness or compassion, like other lines of development, show a classic peak and decline shape—with the peak in early adulthood and decline thereafter (Hartshorne & Germine, 2015)? How are mindfulness and compassion related to other lines of development such as attachment relationships and social-emotional development (Bowlby, 1988; Shaver et al., 2007), self and identity development (Roeser & Peck, 2009; Vago & Silbersweig, 2012), or social-cognitive (Warren et al., 2021) and moral development (Roeser et al., 2014)? What are the biological, social interactional, and sociocultural contextual factors and processes that explain the development and transformation of the elements of mindfulness and compassion over time during childhood and adolescence in families, schools, communities, and religious institutions (Eccles & Roeser, 2016; Semenov & Zelazo, 2019)?

We propose that addressing these unanswered questions regarding a description and explanation of the development of mindfulness and compassion is an important priority in advancing the next generation of science. Specifically, empirical insights and answers to these questions can inform the optimization of programs through the identification of key proximal processes in families and schools that produce mindful and compassionate individuals (or not, e.g., Semenov & Zelazo, 2019). These processes could then be studied within the implementation of SBMPs. Furthermore, such work would advance the conceptualization and measurement of mindfulness and compassion, as well as their antecedents and developmental consequences, across the lifespan. Finally, bringing developmental methods to bear on the study of SBMPs with time-intensive measures like ecological momentary assessments would help us to understand the time course and shape of change in these constructs and related outcomes. Greater cross-fertilization between developmental and experimental research is something that we believe could advance the next generation of science (see Table 3).

## Conclusions

In conclusion, the field of science on school-based mindfulness and compassion programs is still relatively young and growing. The science has shown some promising results, but limitations in the current research, and critiques about the framing, aims, pedagogies, and scientific assumptions in research on mindfulness and compassion in schools require attention. In order to advance science on mindfulness and compassion in schools for students in the next generation, we suggest a need for *re-envisioning* the ways the practices are brought into education, as well as the meta-theoretical assumptions, theories, and methods in this area of research to go “beyond all splits” towards a relationally, culturally, contextually, ethically and developmentally grounded science. We look forward to the coming generation of work aimed at producing generations of students that are more mindful, compassionate, and engaged citizens of the world.

## Supplementary Material

Supplementary Figure S1

## Figures and Tables

**Table 1:** Primary academic discourses in research on mindfulness in education

	Predominant discourse: Mindfulness *in* education	Minor discourse: Mindfulness *as* education

Conceptual *framing* of mindfulness practices	Individualistic Psychological Universal-secular Effects-oriented	Relational Interactional Culturally diverse Process-oriented
Educational *aims* of mindfulness practices	Cognitive skills Social-emotional skills Personal health and Well-being	Awareness Self-knowledge Agency Embodiment Ethics Social justice
Pedagogical *methods* for teaching mindfulness	Extra-educational interventions implemented in schools	Integration into curriculum Whole-school implementation

Summarized from Ergas and Hadar (2019)

**Table 2:** Different meta-theories and root images in the study of mindfulness and compassion

Attributes	Split Meta-Theory	Relational Meta-Theory

Assumptions	Dualistic, either/or Mechanistic Reductionistic	Non-dualistic, both-and Organismic Holistic
Frames	Human/animal Person/context Mind/body Cognition/emotion	Evolved mammal Person embedded in multiple contexts Embodied minds Top-down, bottom-up
Root Images	Clockworks, machines	Cell, living systems

For discussion of meta-models or paradigms in developmental science, see Lerner and Overton (2008); Overton (2007); Sameroff (2010); Zelazo (2013)

**Table 3:** Complementary research aims and methods in experimental and developmental approaches to research on mindfulness and compassion in children and adolescents

Attributes	Experimental approach	Developmental approach

Scientific aims	Preventing psychological problems and reducing psychological suffering	Preventing developmental problems and cultivating developmental competencies
Research aims	Causal impacts of treatment on change in child and adolescent outcomes	Temporal correlations of person-process-context-transactions and change in child and adolescent outcomes
Research methods	Randomized, controlled	Longitudinal, naturalistic
Research timeframe	Pre, post, follow-up	Momentary, lifespan, historical
Hypothesized processes of effect (e.g., “mechanisms”)	Manualized treatments via implementation quality and practice-induced learning of mindfulness skills	Cultural socialization via proximal social processes and caring, attuned relationships with others in shared activity
