# Supplementary material for: Beyond All Splits: Envisioning the Next Generation of Science on Mindfulness and Compassion in Schools for Students
Source: Mindfulness (N Y). Author manuscript; Available in PMC 2025 Jan 31. (PMC11784944; doi:10.1007/s12671-022-02017-z)

Supplementary Figure S1

Google NGram Plot of “Mindfulness in Education” in English Language Books (1990-2019)

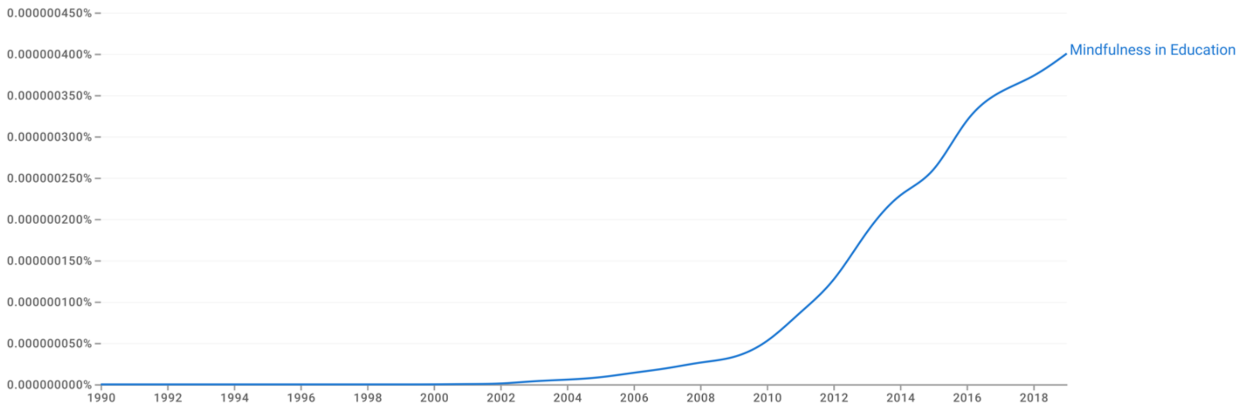

Supplement: Supplementary Figure S1 [file NIHMS2004131-supplement-Supplementary_Figure_S1.pdf]
